# Comparing Eye-Tracking and Verbal Reports in L2 Reading Process Research: Three Qualitative Studies

**DOI:** 10.3390/jemr19010002

**Published:** 2025-12-25

**Authors:** Chengsong Yang, Guangwei Hu, Keyu Que, Na Fan

**Affiliations:** 1 School of Foreign Studies, Xi’an Jiaotong University, Xi’an 710049, China; 2 Department of English and Communication, The Hong Kong Polytechnic University, Hong Kong, China; guangwei.hu@polyu.edu.hk; 3 Beicai Senior High School Affiliated to Shanghai Maritime University, Shanghai 201204, China; quekeyu@163.com; 4 School of Foreign Studies, Shaanxi Normal University, Xi’an 710062, China; fanna@snnu.edu.cn

**Keywords:** eye-tracking, verbal reports, veridicality, reactivity, methodological triangulation

## Abstract

This study compares the roles of eye-tracking and verbal reports (think-alouds and retrospective verbal reports, RVRs) in L2 reading process research through three qualitative studies. Findings indicate that eye-tracking provided precise, quantitative data on visual attention and reading patterns (e.g., fixation duration, gaze plots) and choice-making during gap-filling. Based on our mapping, it was mostly effective in identifying 13 out of 47 reading processing strategies, primarily those involving skimming or scanning that had distinctive eye-movement signatures. Verbal reports, while less exact in measurement, offered direct access to cognitive processes (e.g., strategy use, reasoning) and uncovered content-specific thoughts inaccessible to eye-tracking. Both methods exhibited reactivity: eye-tracking could cause physical discomfort or altered reading behavior, whereas think-alouds could disrupt task flow or enhance reflection. This study reveals the respective strengths and limitations of eye-tracking and verbal reports in L2 reading research. It facilitates a more informed selection and application of these methodological approaches in alignment with specific research objectives, whether employed in isolation or in an integrated manner.

## 1. Introduction

Verbal reports require informants to state their thoughts orally while or after they complete a task, hence the distinction between think-alouds and retrospective verbal reports (RVRs) [1]. Providing access to second language (L2) learners’ and teachers’ minds, verbal reports have long been used to address wide-ranging questions, including the (sub)processes of reading and writing, learner characteristics, learner problems and strategy use, learner responses to feedback, as well as teachers’ feedback giving and rating processes [2,3,4,5]. Eye-tracking records eye movements and typically links the properties of fixations and saccades to cognitive processing or attention allocation [6,7]. Its utilization in applied linguistics research is relatively recent but vibrant and has addressed a number of issues, such as incidental learning, the role of video captions, the cognitive processes underlying test-taking, and dictionary look-up processes [8,9,10,11]. There is a need to compare the distinctive strengths and drawbacks of verbal reports and eye-tracking, as eye-tracking gains increasing popularity. Eye-tracking has limitations, which may not be well known to researchers and teachers in our field, and its constraints may not be transparently reported in publications [12]. We also need to restore confidence in verbal reports, which some may consider an outdated method, by examining their unique affordances in comparison with eye-tracking. Currently, there is a scarcity of research that directly investigates the affordances and limitations of both eye-tracking and verbal reports.

Given this scarcity, the present study set out to compare eye-tracking and verbal reports (think-alouds and RVRs) in L2 reading process research. We adopted a purely qualitative approach, conducting three studies to explore eye-tracking’s roles in reading strategy research relative to verbal reports, to compare their affordances in revealing gap-filling processes, and to examine their reactivity as perceived by participants.

### 1.1. Pros and Cons of Verbal Reports and Eye-Tracking

Think-alouds are oral self-reports that reflect the heeded information in working memory that is ‘‘relatively stable and can thus be input to a verbalization process’’ [1] (p. xiii). In comparison, eye-tracking is visually based and, as Holmqvist et al. [13] note, offers an “information source on perceptual/attentional processes” (p. 95). As evidence of cognitive processes, think-alouds have been described as “one of the most direct … methods to gain information about subjects’ internal states” [14] (p. 220), including “the reasoning processes underlying higher level cognitive activity” [2] (p. 308). Eye movements may be considered indirect evidence of cognitive processes, and additional information is indispensable for sound inference, such as determining whether a succession of fixations reflects summarizing or comparing processes.

Think-alouds are often questioned for reactivity and veridicality. Reactivity is the alteration of thinking due to the method used to elicit it. Veridicality refers to the provision of incomplete or inaccurate information, or “errors of omission” and “errors of commission” [15] (p. 760). Think-alouds are likely to cause reactivity due to the requirement to verbalize thoughts concurrently. The reactivity of think-alouds has been considered to be “dependent on a host of variables” in applied linguistics [16] (p. 110). Think-alouds affect different language tasks, for example, reading comprehension, incidental vocabulary learning, and L2 writing, differently [17,18]). Regarding the veridicality of think-aloud protocols, Ericsson and Simon [1] argue that think-alouds are not subject to this concern, as the protocols are able to include what is verbalizable, which is what is heeded. However, think-alouds should not be regarded as veridical if processes that are not verbalizable, for example, “retrieval and recognition processes” [1] (p. xiii), are to be explored [19]. Research has shown that raters or L2 learners failed to tell or refrained from telling certain thoughts concurrently or thought aloud with some deviation (e.g., those that flashed by or concerned privacy) [20,21]. RVRs do not cause reactivity but may be problematic in terms of veridicality due to memory decay over time.

By contrast, eye-tracking is often credited with non-intrusiveness [22]. However, eye-tracking may not be free from reactivity, especially when subtle effects such as participants’ awareness of being eye-tracked are considered [17]. Veridicality is generally not regarded as an issue for eye-tracking, given its objectivity and millisecond-level temporal resolution. However, certain attentional processes may not be captured by eye fixations alone (e.g., use of advance information via parafoveal vision) [23]. Data loss can also occur due to blinks, head movements out of the tracker’s range, or poor calibration, resulting in temporal gaps and biased sampling. Attention should also be paid to the accuracy of eye-tracking, which can restrict its application to language studies, where texts are dense and research may focus on specific parts of words. High-end research eye-trackers, such as EyeLink 1000 Plus and Tobii Pro Spectrum, are specified to achieve a tracking accuracy of approximately 0.3° of visual angle. This accuracy is often reduced in real-world research scenarios due to participants’ inability to maintain a stable gaze and occurrences of head or body movement, which causes calibration drift. Calibration drift is the slow shift of the calculated point of gaze from its true location over the course of an experiment, posing a threat to data accuracy.

### 1.2. Studies Comparing Think-Alouds and Eye-Tracking

Two studies are reviewed here that involve dedicated comparison of think-alouds and eye-tracking. Williams and Davids [24] compared think-alouds and eye-movement data collected to measure soccer players’ selective attention as the players watched match simulations to judge the direction of the final pass of the ball. They found that the two methods were similarly able to reveal the areas of the screen the players paid attention to as the offensive play went on. However, they also reported that think-alouds could not catch as much attentional shift in dynamic situations but were better able to register attention to information outside central vision, which eye-tracking did not record. Godfroid and Spino [17] conducted an experimental study on the reactivity of think-alouds and eye-tracking in researching L2 reading and incidental vocabulary learning. They found that for advanced learners of English, think-alouds and eye-tracking were similarly nonreactive to reading, as neither affected text comprehension. However, thinking aloud was found to facilitate vocabulary recognition and appeared to be more reactive than eye tracking. To our knowledge, no studies have yet compared the roles of verbal reports and eye-tracking and their reactivity by eliciting informants’ views in L2 reading process research.

## 2. Materials and Methods

We designed a series of three qualitative studies comparing the roles of verbal reports and eye-tracking and examining their reactivity. In Study 1, we aimed to explore what eye-tracking could contribute to reading strategy research, in comparison to verbal reports. We adopted Anderson’s categories of 47 reading processing strategies [3], which were developed from RVRs and built on Pritchard’s taxonomy (derived from self-paced introspection and explanatory retrospection) and Nevo’s checklist of test-taking strategies [25,26]. We exercised expert judgment to assess the extent to which eye-tracking data could be used to identify each strategy. The first author made initial independent judgements. He then conducted paired discussions, first with a colleague specialized in eye-tracking research, and subsequently with the fourth author; both had been asked to make their judgments and provide the rationale. In these sessions, we deliberated on whether each of the 47 strategies could be directly identified (i.e., without relying on supplementary methods such as verbal reports) through the examination and analysis of eye movement patterns or statistics. After the discussions, the first author reviewed the audio recordings to consolidate the viewpoints and produced a finalized list. This list was shared with the other two discussants, both of whom confirmed they had no objections.

In Study 2, we examined the pros and cons of eye-tracking and verbal reports. Seventeen, nine, and ten first-year university students were asked to complete two banked gap-filling L2 reading tasks (see Appendix A) under the eye-tracking (plus RVRs), think-aloud, and online immediate RVR (reporting thoughts regarding each choice immediately after it was made) conditions, respectively. The two tasks (“Tower” and “Sara”) were selected from China’s College English Test Band-4 and Band-6 resources. Participants were to select the most appropriate word from the bank for each of the ten blanks. While participants were randomly assigned to the three conditions, we intentionally oversampled the eye-tracking group to gather more student perspectives on eye-tracking’s reactivity for Study 3. Participants were recruited via advertisement, following ethics approval. Background information on the 36 students, including age, gender, and proficiency, is presented in Table 1. Eye-tracking was conducted in our lab with a Tobii TX300 eye-tracker (Tobii, Stockholm, Sweden). All data were collected in individual sessions. After a briefing and the signing of the informed consent form (which covered rights to withdraw, compensation, etc.), each participant underwent training and practice prior to their task. The formal procedure began after their readiness was confirmed. No signs of discomfort were noted during data collection, despite the reactive effects they later reported. While the eye-tracking group made selections by clicking the flip menu located in each blank while reading the test on the computer screen, participants in the verbal reporting conditions completed the tasks using a paper-based test booklet and pen. There was no time constraint. We compared the types of information collected via the three methods on participants’ reading and gap-filling processes. In analyzing the eye-movement data, heatmaps and gaze plots were repeatedly replayed to determine the inclusion and exclusion of fixation points. Timestamped mouse clicks were also employed to clarify the temporal dynamics of the choice-making processes.

In Study 3, which focused on reactivity, we analyzed the answers of the 36 participants from Study 2 to follow-up open-ended questionnaires. Each questionnaire asked whether and how the method they experienced affected their reading and gap-filling processes and correctness. We followed Thomas’s inductive approach to coding [27]. After his initial work, the first author worked with another author inductively in multiple rounds of independent coding until finally the agreement rates for the categories extracted (2 × number of identical codes/number of total codes, counting all coding discrepancies) all reached above 80% (e.g., 81.82% for the causes of eye-tracking’s reactivity, Kappa = 0.776; 80.77% for the effects of eye-tracking, kappa = 0.748). All discrepancies were then discussed and resolved (see Appendix B for our codebook and examples of coding). In our coding of the reactivity of eye-tracking, we excluded a number of participants’ reports about their maladjustment to computerized test-taking, most notably their being unable to take notes.

## 3. Results

In Study 1, although Anderson’s [3] list of 47 processing strategies was developed from verbal reports, we jointly ascertained that only 13 strategies could be confidently identified via eye-tracking. These strategies are listed in Table 2 (see Anderson [3], p. 463, for the full list of reading processing strategies). All the supervising strategies (e.g., making predictions), paraphrase strategies (e.g., using cognates), and others (e.g., needing a dictionary, visualizing, using background knowledge, reasons for choice, and assessment of correctness) did not make their way to Table 2. We agreed that these strategies involved specific contents of thinking that could either only be accessed with verbal reports (e.g., reasons for a choice), or only be partially captured by eye-tracking. For example, regarding our exclusion of the first supervising strategy in Anderson’s [3] list, “refers to the experimental task,” the first author and his colleague envisioned scenarios where readers might make regressions—that is, look back at the instructions—when they needed that information for supervising (e.g., to stick to the goal of reading, to solve problems) but forgot it or wanted to confirm it. However, while we recognized eye-tracking’s roles, we also agreed that readers might use information from the experimental task to guide their reading without rereading it; eye-tracking evidence might not be sufficient in such cases. As an illustration of our inclusion, we all agreed that the test-taking strategy of “looking for answers in chronological order” could be identified from eye movement patterns. This was because we thought we were able to examine whether a test-taker completed the questions in the order they appeared in the text, which would be reflected in a sequential reading path closely following the passage’s structure. It should be noted that identifying some of the included strategies required the screen recording function of the eye-tracker, in addition to eye movements, and this process would be rather time-consuming.

Given the difficulty of deriving general comparisons from the variety of reports and eye-movement features, we present a case illustration from Study 2. We focused on the completion of a single blank, Blank 7 (“to have a national holiday declared that would bring families together while celebrating the _____ festivals”), which was the easiest, with 35 out of the 36 participants answering correctly. We compared the process information for this item from a high scorer P6 (female, *gaokao* English score 147 out of 150) based on her eye-tracking, a low scorer P19 (male, *gaokao* English score 109) based on his think-alouds, and 10 participants (P27 to P36) based on their online immediate RVRs.

Table 3 presents the types of information that eye-tracking can offer: (1) a general picture of visual processing shown in a heatmap, (2) exact records of the time course and procedure of choice making, which can be visualized in a graph, (3) measures such as fixation duration and visits, and (4) a gaze plot, which depicts the fixation path. Such information leads to the conclusion that Item 7 was completed expeditiously and without much effort, confirming its ease. The data suggest that P6 may have initially interpreted the syntactic structure as requiring a noun after “the”, but upon reading “festivals”, she corrected her interpretation and switched to searching for an adjective, which led directly to the final answer. It should also be noted that it was only from P6’s statements in her offline RVRs (“traditional fes-, festivals [mispronunciation] … *It is traditional festival*”) that we know this high scorer arrived at the answer largely based on her understanding of the two adjacent words.

Table 4 presents the information elicited from think-alouds and online immediate RVRs. The think-alouds on Blank 7 include two accounts from P19, which contain information on time, frequency of mentioning, and procedural details (e.g., time spent on the item, mentions of words in adjacent text, checking times, and non-revision). These accounts could be coded to analyze the learner’s use of strategies and textual information. They reveal the processes by which the low scorer was able to achieve the correct answer quickly, similar to the high scorer. Admittedly, compared with eye-tracking, think-alouds were incomplete (e.g., Episode 1’s failure to capture P19’s rapid search for “traditional” within the bank), and reading processing calculated based on the length of utterances and mentions was not as fine-grained and accurate. For example, it remained unknown whether P19 processed the adjacent text when he remarked on his answer in Episode 2. However, think-alouds uniquely made explicit the low scorer’s strategies and information use (e.g., part-of-speech analyses), as well as his attempts to translate, frequent recognition, guessing, and misinterpretations of words unknown (e.g., mistaking “lamb” for “lame”), thereby opening other research venues, such as L1 use in L2 reading.

Table 4 also presents online immediate RVRs, from which strategy and information use can be extracted. Most participants demonstrated knowledge of the formulaic phrase “traditional festivals” and made assertions that “traditional” should be the answer (e.g., P32, P27, P28 and P30), was the answer (P34, P36), or “*generally*” was the answer (P31). A couple of participants referred to some previous text, such as P28’s mention of a key word, “Thanksgiving”. The online immediate RVRs did not yield as much process information as the think-alouds.

In Study 3, four key themes emerged from the informants’ answers: (1) whether any effects, negative or facilitative, were mentioned or implied, (2) the extents of these effects, (3) their causes, and (4) the aspects of reading and banked gap-filling that were affected. The first theme comprised four codes: (1) “positive,” (2) “negative,” (3) “positive with negative,” and (4) “no influence.” The second theme included four levels: (1) “very big,” (2) “fairly big,” (3) “some,” and (4) “small.” As for the causes of reactivity, the third theme identified five broad factors related to eye-tracking: (1) “stabilized head,” (2) “awareness of eye-tracking,” (3) “interference from hardware,” (4) “answering by clicking on a flip menu,” and (5) “being in an experiment.” Three causes of verbal reports’ reactivity were derived: “verbalization,” “counter-habitual behavior,” and “voice as noise.” Under the fourth theme, five general subcategories were developed regarding the specific effects of eye-tracking: (1) “physical, emotional, or psychological effects,” (2) “the way they looked or read,” (3) “distraction,” (4) “correctness,” and (5) “speed.” Additionally, four specific aspects of influence emerged from the verbalizers’ accounts: “processes,” “speed,” “correctness,” and “nervousness.”

**Figure 1:**
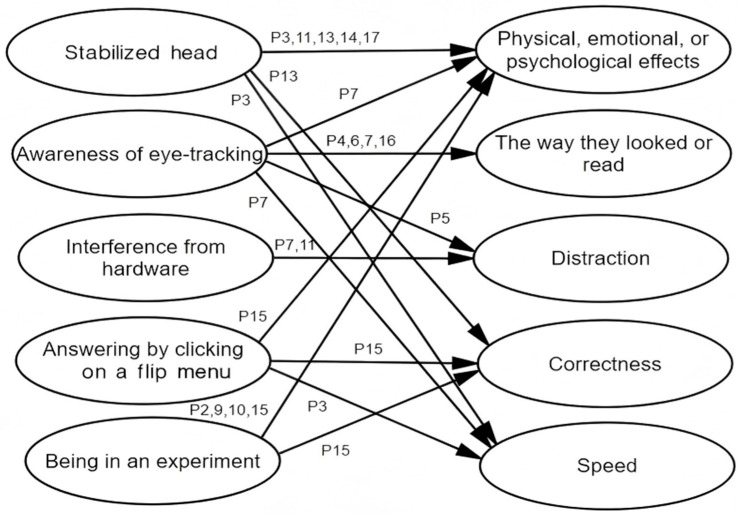
Causes and effects of reactivity of eye-tracking.

Among the 17 participants who were eye-tracked, 13 reported being negatively affected, but 12 of them mitigated these effects with modifiers (e.g., “*small,*” “*not big,*” “*somewhat,*” or “*to some extent*”) or limited the situation (e.g., their maladjustment) to the initial phase of the experiment. Four participants denied being affected. The causes and effects are linked in Figure 1.

Having the head stabilized or maintaining a stable posture was the most noticeable cause, reported by five participants, which was a high number considering that a chin rest was purposively applied to nine of the 17 participants. This factor mainly caused physical, emotional, and psychological effects. For example, P13, who spent about 27 min and answered 20% of the questions correctly, described a chain of effects: “*a sore neck,*” “*some loss of attention,*” “*more anxiety,*” “*failure to analyze some sentences calmly,*” and reduced accuracy. Awareness of eyes being tracked was also reported as a cause by five participants. It first influenced the way they looked or read. P6, for example, wrote, “*aware of being eye-tracked, I tried not to stay too long on a sentence or a word, not to look away from the screen, not to look elsewhere and think for a long time.*” (see Appendix C for the handwritten answer of P6). P4 mentioned that, but for eye-tracking, she would have tried to recall in her mind words that had appeared, instead of intentionally reading them again. P7 noted that awareness of the eye-tracking caused him to read more slowly and with a more restricted range of eye movements. P16 also reported, “*I would think I was being eye-tracked. This affected my line of sight and how I read (approached) the questions while completing the task*.” For P5, such awareness became a distraction, making him pay attention to his own eye movements. This awareness had other effects on P7, who mentioned, most notably, a facilitative effect (i.e., becoming more focused), in addition to psychological pressure. Being in an experiment was a source of reactivity for four participants, causing mostly physical, emotional, and psychological effects (e.g., nervousness). The hardware disturbed two participants: for P11, the distraction was the “*red circle*” (illuminator) of the eye-tracker, and for P7, the noise of the “*machine operation*”) (see Appendix C for the handwritten answers of P7). The final cause was the unaccustomed answering method, designed to facilitate eye-movement data analysis. Figure 1 also shows that physical, emotional, and psychological effects were reported by 10 participants, followed by changes in the way they looked or read, which was reported by four participants.

Eight of the nine participants who performed think-alouds reported feeling negatively affected, whereas two of these eight also mentioned facilitative effects, and one reported only facilitative effects. Reflections from six think-aloud participants revealed varying extents of these effects. Except for P25, who claimed a “*pretty big*” effect on correctness, five felt affected only to a small extent or to some extent. Interestingly, P21 described the effects as dynamic, becoming weaker as he became immersed in the task, but occasionally rebounding when he became distracted or nervous, especially when he had nothing to verbalize due to difficulty finding an answer. Most notably, seven participants felt that their reading and gap-filling processes were altered or disrupted. Five of these seven stated that negative effects were caused by having to divide their attention for verbalization (e.g., P21) or by perceiving their own voice as a source of noise (P25). Three participants reported facilitative effects, though one of them (P22) also reported a negative effect. P18 felt more clear-minded, while P26 felt more thoughtful, rational, and less dependent on intuition. P22 elaborated: “*It strengthens the thinking process of brain circuits, makes the thinking more complete, and forms a secondary memory with the visual information.*” In addition, six of the nine participants mentioned that thinking aloud was unusual or counter to their habits, and five thought that it slowed their performance. Five participants expressed concerns about reduced correctness. Finally, only P21 mentioned experiencing nervousness.

Among the 10 participants who provided online RVRs, four reported no effects, five mentioned positive effects (with three of these also mentioning negative effects), and one reported only negative effects. Two participants described the effects as being slight. Six participants described the effects in terms of alterations to their thinking, reading, or gap-filling processes. Of these six, two (P28 and P29) mentioned a disruptive effect, while five (including P28) reported facilitative effects. Online RVRs prompted some participants to think twice (P30), or made them feel more clear-minded, lively-minded, and/or less reliant on intuition (P28, P33, P34, P35, and P36). Consequently, three participants (P28, P33, and P35) mentioned the possibility of increased accuracy. Finally, three participants (P28, P30, and P33) thought that online RVRs slowed them down.

## 4. Discussion

In Study 1, we agreed that eye-tracking played a limited or complementary role in identifying reading processing strategies. In reading strategy research, verbal reports have long been collected for easy, direct access to learners’ thoughts about what they do, how, and why they do it to solve problems, which are then coded as various strategies. These strategies are not limited to reading patterns that are identifiable through ocular data. Our findings thus support the unique benefit of verbal reports as an easy, direct means of accessing the human mind [2,7] and highlight the limitation of eye-movement data, namely their indirectness [13].

Study 2 demonstrated the variety of process information on reading and gap-filling that eye-tracking can offer, featuring fine-grained data, exact quantification, and easy visualization. Think-alouds provided similar types of reading processing and procedural information, indicating that depending on the research question, think-alouds and eye-tracking may serve the same purpose [24]. However, think-alouds lacked the level of measurement exactness that eye-tracking could achieve and was insufficiently complete when it came to transitory processes that were difficult to verbalize [1,13]. The study also illustrated that online immediate RVRs could collect learners’ thoughts directly, but such verbal reports did not yield as much process information as the think-alouds.

Study 3 revealed the subtle effects of eye-tracking and their specific causes. These effects were mostly derived from causes characteristic of eye-tracking (i.e., head stabilization, hardware, and format change) and primarily resulted in physical, emotional, and psychological effects. These findings enrich Godfroid and Spino’s summary on the reactivity of eye-tracking [17]. Study 3 also revealed the multi-faceted effects of think-alouds. Think-alouds were most often reported to cause alteration or disruption of thinking and were considered an unusual way of completing the reading task. These findings differ from those of Godfroid and Spino and others, who inferred non-reactivity from performance indexes [17]. This difference should not be surprising, as Barkaoui [13] has reported similar feelings among teacher raters regarding the validity of think-alouds. Understandably, online RVRs were viewed as less disruptive, as they required only intermittent reports during task completion. Compared with eye-tracking, both types of verbal reports could engender facilitative effects, such as feelings of being clear-minded and less intuitive, as well as the benefit of memory enhancement, with those of online RVRs reportedly more prominent. In fact, both Russo et al. [15] and Stratman and Hamp-Lyons [29] have summarized the facilitative effects of concurrent verbal reports, which may be caused by verbalizing thoughts and listening to one’s own voice, in terms of enhanced memorization and learning via reflection. In L2 research, such effects have also been extensively documented and discussed [16,30,31]. Online RVRs may have offered a particular opportunity for reflection. It should be noted that while the reactive effects of think-alouds are considered a methodological confound to be minimized in research, the facilitative effects of both types of verbal reports reveal significant potential for application in educational settings [16,32]. In practices such as process diagnosis and teaching intervention, this facilitation can be reconceptualized as a targeted tool to enhance learning. The reactivity studies also show that being in an experiment or being studied may cause some reactivity, which may apply to both eye-tracking and verbal reports. As a final note, although eye-tracking, think-alouds, and online immediate RVRs may incur reactive effects, as reported by the participants, their value in eliciting the process of thinking is undeniable. In L2 reading research, the sacrifice of some ecological validity is often a justified and essential step toward a deeper, more authentic understanding of the reading process.

## 5. Conclusions

Although their roles may overlap in gauging attention or language processing, eye-tracking and think-alouds are different sources of information and have their respective “niches.” Eye-tracking offers fine-grained information on visual activities and is a superior method for studies involving reading and viewing where exact qualification is required and transitory cognitive processes are of interest. Verbal reports provide easy, direct access to thoughts, making them irreplaceable where researchers need precise insight into learners’ thinking, such as in research on L2 reading strategies. Both verbal reports and eye-tracking can cause some subtle effects. These effects may be specific to these methods or may be common to any research method in a study setting. We do not consider these effects to be serious, and we believe that they can be minimized. The current popularity of eye-tracking notwithstanding, we call for a more balanced view of think-alouds and renewed recognition of the unique benefits of verbal reports. We advocate for a principled approach to methodological selection, maintaining that researchers should align their choice with specific research objectives—employing either method in isolation or integrating them to leverage their complementary strengths. For example, in eye-tracking study on L2 reading processes, the benefits of verbal reports may be exploited by including a think-aloud case study or even a parallel think-aloud group, in addition to follow-up RVRs, to yield a more comprehensive understanding.

Our case studies have limitations. First, although the findings of Study 1 were based on discussions among two authors and a colleague and eye-tracking researcher, the results were still subjective. However, we maintain that the purpose of presenting these preliminary findings is not to offer a definitive conclusion, but to stimulate further scholarly inquiry. Second, in Study 2, the comparative results in our illustration should be generalized with caution. This is because the content of think-aloud and RVR protocols varies across individuals, being influenced by multifarious factors such as verbosity and proficiency, while eye-movements are themselves highly individualized and contextualized. Third, Study 3 would benefit from recruiting more participants to achieve a fuller understanding of the reactivity of eye-tracking. Participants’ self-perceived effects should be triangulated with performance changes (including eye-movement changes) under their particular conditions. The reactive effects reported by participants on increased or reduced accuracy, in particular, may not be real and require further validation. We therefore propose that larger-scale studies incorporating stricter quantitative analyses be conducted to explore the separate and complementary roles of eye-tracking and think-alouds and to compare their reactivity. Finally, although we employed strict measures to enhance the validity of our eye-movement data and present only one participant’s eye-movement data and the results of our analyses, issues inherent to eye-tracking, including data loss and calibration drift, may influence the accuracy and generalizability of our results.

## Figures and Tables

**Table 1:** Background information on participants.

Condition	*n*	Age		Gender		*Gaokao* English Score				Gap-Filling Scores			
		*M*	*SD*	M	F	*M*	*SD*	Min.	Max.	*M*	*SD*	Min.	Max.
Eye-tracking	17	18.80	0.69	8	9	129.24	21.90	85	148	51.47%	0.28	15%	95%
Think-alouds	9	18.74	0.42	6	3	135.56	16.41	105	146	48.89%	0.21	20%	75%
RVRs	10	18.46	0.32	8	2	127	22.78	85	146	51.5%	0.25	10%	80%

Note. *Gaokao* is the national university entrance exam, and its full English score is 150.

**Table 2:** Processing strategies most helpfully identified via eye-tracking.

II Support strategies	“skims reading material for a general understanding”
	“scans reading material for a specific word or phrase”
IV Strategies for stablishing coherence	“rereads”
	“reads ahead”
V Test-taking strategies	“looks for the answers in chronological order in the passage”
	“matches the stem and/or alternatives to a previous portion of the text”
	“reads the questions and options after reading the passage”
	“reads the questions and options before reading the passage”
	“changes an answer after having marked one”
	“stops reading the options when they reach the answer”
	“skips a question and returns to it later”
	“skips a question that is not understood and leaves the response blank”
	“marks answers without reading in order to fill the space”

**Table 3:** Information from eye-tracking (from P6, with 90% accuracy on the task).

(1) Heatmap: The heatmap reveals that Blank 7 (circled) was not intensely processed, compared with other blanks such as Blank 10, the last one, which attracted a much greater accumulation of fixations, as indicated by several prominent yellow clouds.
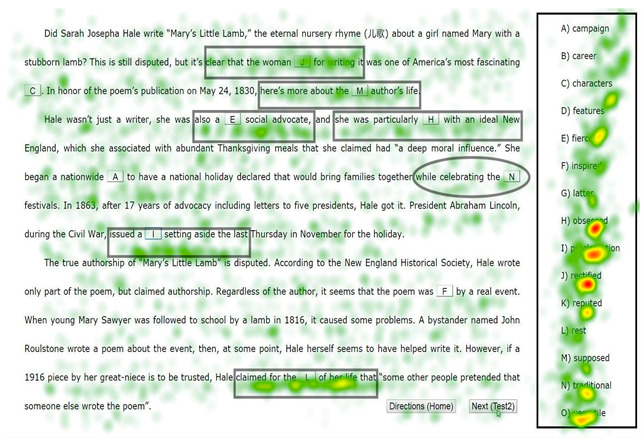
(2) Choice-making graph: The choice-making graph demonstrates a straightforward selection for Blank 7 (the circled “N” choice, completed at 04:35), which was made in a single attempt without revision. In contrast, the final selection of “L” for Blank 10 was preceded by four tentative choices and considerable hesitation. The graph also shows that the completion of Blank 7 followed the first closure of Blank 6 and was followed by the completion of Blank 9, skipping over Blank 8.
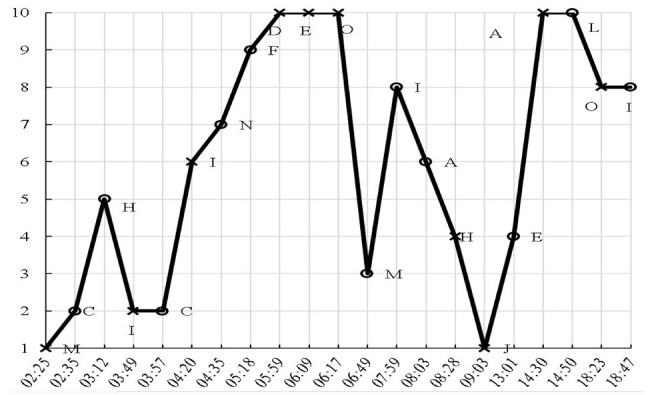
(3) Measures such as fixation duration, fixation counts, and visits: 14,788 ms was spent completing Blank 7; A total of 38 fixations were recorded during this completion; The total fixation duration (TFD) was 1120 ms on the adjacent text (“while celebrating the”, “festivals”), 4480 ms inside the bank, and 1380 ms on the option “traditional”; The item was revisited twice (triggered by “versatile” and “traditional”).
(4) Gaze plot:
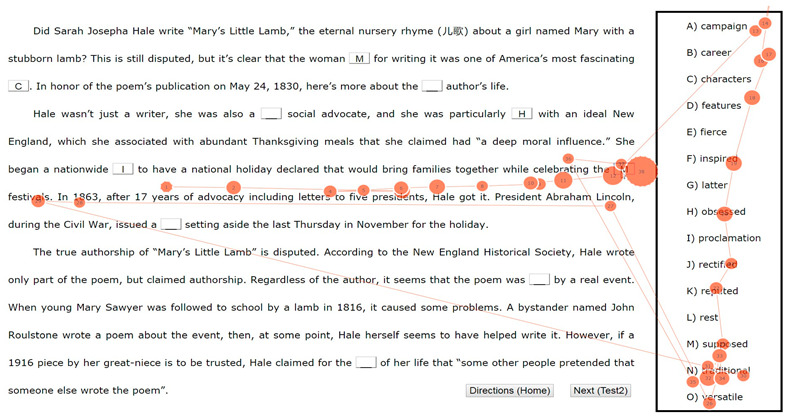
The gaze plot shows precisely what P6 read and her reading sequence during the completion of Blank 7. The plot uses red dots to represent fixations, with numerical labels indicating their sequence and dot size corresponding to fixation duration. As shown, P6 read the text before and after the blank, as well as a wide array of options within the bank. Fine-grained analyses reveal that P6 first read the text preceding the blank, starting from the word “have”. Upon reading the word “the” to the left of the blank, she began to read the options. Her scan began at the top with “campaign” and proceeded downward through the options to “versatile.” She then returned to the word “festivals” following the blank, which was the final word of the sentence (after a brief fixation on “Lincoln”). At this point, she may have realized that an adjective, rather than a noun, was required. She then returned directly to fixate on the option “traditional” before finalizing her choice in the blank. (5) Other relevant information that may be gained via replay of screen recording: The whole passage, including Blank 7, was previewed; The answer was checked 8 times.

**Table 4:** Information from think-alouds and online immediate RVRs.

Think-alouds (from P19, who achieved 10% accuracy on the task)	Online immediate RVRs (from P27 to P36, who achieved 0 to 80% accuracy on the task)
Two episodes concerning Blank 7 in P19’s think-alouds: “that would bring fa- families (pause), declared that (pause) would bring families, together while celebrate the *what what* festivals. *Should be a, should be a beautiful, a beautiful adjective. Oh, no, Thanksgiving is a traditional festival, so it should be* traditional. *It should be* traditional festivals” (38 s). “… the *what* festivals, the traditional festivals, *this, this blank, I am sure, am sure this N should be chosen*” (16 s). Temporal measures and times of mentioning calculated based on the think-alouds: (1) Blank 7 was solved within 54 s, compared with, e.g., Blank 1, which was finished in four episodes lasting 2 min and 48 s; (2) processing time on adjacent text 10 s, and on *traditional* about 3 s; (3) *traditional* was mentioned four times, and words in adjacent text were mentioned a total of 11 times. Choice-making Blank 7 was completed when first met (during Episode 1); The answer was checked once but not revised (during Episode 2); Strategies: predicting the meaning of the deleted word, conducting part-of-speech analysis, referencing a previous word, making choice based on understanding. Use of information: within clause and extra-sentential. His answer paper: 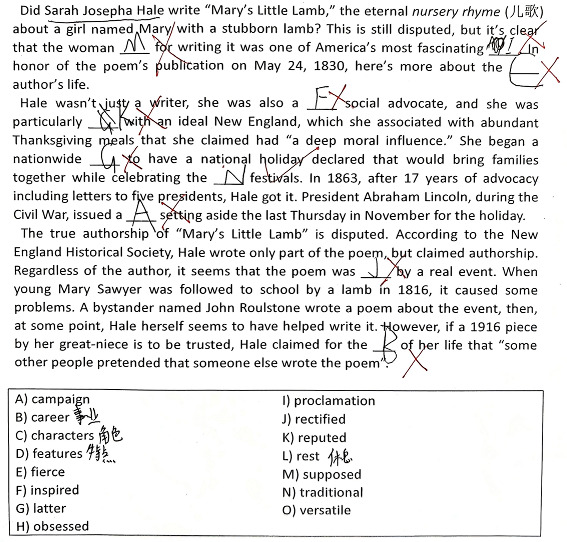	P32 (80% accuracy): “while celebrating the (.) *I guess it should be festivals*, the traditional festivals.” P33 (80% accuracy): “would bring, *that is, get the family together to celebrate this traditional festival.*” P27 (70% accuracy): “*Celebrating the what festivals, must be celebrating the traditional festivals, that is, celebrating this traditional festival,* would bring families together, *that is, make families cohesive.*” P28 (70% accuracy): “*Next, I should choose a word to collocate with this* festivals. *Based on my experience, and the reference to this foregoing* Thanksgiving, traditional *should be chosen.*” P30 (70% accuracy): “*The 7th blank,* traditional festival *should be chosen.*” P31 (50% accuracy): “*Because generally it is celebrating some traditional festivals.*” P34 (40% accuracy): “*It is* traditional festivals, *the previous choice was wrong.*” P36 (20% accuracy): “*It also is a* traditional.” P29 (0% accuracy): “*This blank is celebrating what kind of festival. Then, down below many words I don’t know. I will then choose* L” (*rest*). P35 (60% accuracy): Recording missing. Their answer papers were also available.

Note. Strategies based on Anderson [3], and information use on Bachman [28]. Italics in quotations indicate translation.

## Data Availability

All necessary data has been presented in the article and the full data can be made available on request.

## Abbreviations

The following abbreviations are used in this manuscript:

RVRs	Retrospective verbal reports
L2	Second Language

## Appendix A. The Two Gap-Filling Tasks

### Appendix A.1. The “Tower” Task

An office tower on Miller Street in Manchester is completely covered in solar panels. They are used to create some of the energy used by the insurance company inside. When the tower was first (1)____ in 1962, it was covered with thin square stones. These small square stones became a problem for the building and continued to fall off the face for 40 years until a major renovation was (2)____. During this renovation the building’s owners, CIS, (3)____ the solar panel company, Solarcentury. They agreed to cover the entire building in solar panels. In 2004, the completed CIS tower became Europe’s largest (4)____ of vertical solar panels. A vertical solar project on such a large (5)____ has never been repeated since.

Covering a skyscraper with solar panels had never been done before, and the CIS tower was chosen as one of the “10 best green energy projects”. For a long time after this renovation project, it was the tallest building in the United Kingdom, but it was (6)____ overtaken by the Millbank Tower.

Green buildings like this aren’t (7)____ cost-efficient for the investor, but it does produce much less pollution than that caused by energy (8)____ through fossil fuels. As solar panels get (9)____, the world is likely to see more skyscrapers covered in solar panels, collecting energy much like trees do. Imagine a world where building the tallest skyscraper wasn’t a race of (10)____, but rather one to collect the most solar energy.

(A) cheaper	(I) eventually
(B) cleaner	(J) height
(C) collection	(K) necessarily
(D) competed	(L) production
(E) constructed	(M) range
(F) consulted	(N) scale
(G) dimension	(O) undertaken
(H) discovered	

### Appendix A.2. The “Sara” Task

Did Sarah Josepha Hale write “Mary’s Little Lamb,” the eternal nursery rhyme (儿歌) about a girl named Mary with a stubborn lamb? This is still disputed, but it’s clear that the woman (1)____ for writing it was one of America’s most fascinating (2)____. In honor of the poem’s publication on 24 May 1830, here’s more about the (3)____ author’s life.

Hale wasn’t just a writer, she was also a (4)____ social advocate, and she was particularly (5)____ with an ideal New England, which she associated with abundant Thanksgiving meals that she claimed had “a deep moral influence.” She began a nationwide (6)____ to have a national holiday declared that would bring families together while celebrating the (7)____ festivals. In 1863, after 17 years of advocacy including letters to five presidents, Hale got it. President Abraham Lincoln, during the Civil War, issued a (8)____ setting aside the last Thursday in November for the holiday.

The true authorship of “Mary’s Little Lamb” is disputed. According to the New England Historical Society, Hale wrote only part of the poem, but claimed authorship. Regardless of the author, it seems that the poem was (9)____ by a real event. When young Mary Sawyer was followed to school by a lamb in 1816, it caused some problems. A bystander named John Roulstone wrote a poem about the event, then, at some point, Hale herself seems to have helped write it. However, if a 1916 piece by her great-niece is to be trusted, Hale claimed for the (10)___ of her life that “some other people pretended that someone else wrote the poem”.

(A) campaign	(I) proclamation
(B) career	(J) rectified
(C) characters	(K) reputed
(D) features	(L) rest
(E) fierce	(M) supposed
(F) inspired	(N) traditional
(G) latter	(O) versatile
(H) obsessed	

## Appendix B. Codes and Examples

### Appendix B.1. Reactivity of Eye Tracking

Theme 1: whether any effects, negative or facilitative, were mentioned or implied.

Code (1) “positive,” (2) “negative,” (3) “positive with negative,” and (4) “no influence”.

Theme 2: the extents of these effects.

Code (1) “very big,” (2) “fairly big,” (3) “some,” and (4) “small”.

Theme 3: their causes.

Code (1) “stabilized head,” (2) “awareness of eye-tracking,” (3) “interference from hardware,” (4) “answering by clicking on a flip menu,” and 5) “being in an experiment”.

Theme 4: the aspects of reading and banked gap-filling that were affected.

Code (1) “physical, emotional, or psychological effects,” (2) “the way they looked or read,” (3) “distraction,” (4) “correctness,” and (5) “speed”.

e.g., P7:

(1) 有影响 (Theme 1, Code 2), 听到了一些机器运行的声音 (Theme 3, Code 3), 尤其在我跨越较大或读完一行开始读下行时. 这种声音的突变在做第一篇文章时表现较明显. 他仿佛在提示我重新回去读上一秒在阅读的东西 (Theme 4, Code 3).

[Translation: “(1) *Affected. I could hear some machine operation sounds, especially when I moved my gaze over a large distance or when I finished reading one line and started the next. This variation in sound was more noticeable during the first article. It seemed to prompt me to go back and reread what I had read a second ago.*”]

(2) 没大影响 (Theme 2, Code 4). 只是被告知有跟踪时 (Theme 3, Code 2), 会不自觉的产生心理压力 (Theme 4, Code 1) 并在阅读时减缓了速度 (Theme 4, Code 5) 且减少了活动范围 (Theme 4, Code 2), 较高度的精力集中使我在每一篇文章的末尾已有些累了 (Theme 4, Code 1).

[Translation: “(2) *No major impact. Except that being told about the tracking unconsciously created psychological pressure, leading me to lower my reading speed and reduce my range of eye movements. The heightened concentration made me feel somewhat fatigued by the end of each article.*”]

(Overlapping codes are counted once only.)

### Appendix B.2. Reactivity of Think-Alouds and RVRs

Theme 3: their causes.

Code (1) “verbalization,” (2) “counter-habitual behavior,” and (3) “voice as noise”.

Theme 4: the aspects of reading and banked gap-filling that were affected.

Code (1) “processes,” (2) “speed,” (3) “correctness,” and (4) “nervousness”.

e.g., P19 (Think-alouds):

(Theme 1, Code 2) 由于之前做题只是在脑子里想, 所以一开始边说边做有点 (Theme 2, Code 4) 不适应 (Theme 3, Code 2), 再加上感觉这些题比较难, 所以觉得边说边做有一点 (Theme 2, Code 4) 吃力 (Theme 3, Code 2), 在说的过程中因为是根据自己的思路, 所以说出来的有时候会含糊不清, 可能是因为不适应的原因 (Theme 3, Code 2), 我觉得如果与不说相比较的话, 说出来的正确率不会比不说要高 (Theme 4, Code 3), 而且速度比较慢 (Theme 4, Code 2).

[Translation: “*As I usually just think through the questions in my head when working on them, speaking out loud while solving them felt a bit awkward at first. Also, since the questions seemed difficult to me, I found it somewhat strenuous to speak and solve them at the same time. When I spoke, since I was following my own train of thought, what I said sometimes came out unclear, maybe because I wasn’t used to it. Compared to not speaking aloud, I don’t think speaking would lead to higher accuracy, and it also felt slower*.”]

P34 (RVRs):

(1) 不影响, 只是很脑海里想的, 我口述表达不出来.
(2) 不影响, 口述 (Theme 3, Code 1) 甚至让我脑海更清楚, 让我对自己的做题过程更加深刻 (Theme 1, Code 1) (Theme 4, Code 1).

[Translation: “(1) *No effect. It’s just that what I think in my head, I couldn’t quite articulate it verbally.* (2) *No effect. Verbalizing even made my thoughts clearer and gave me a deeper understanding of my own problem-solving process.*”]

## Appendix C. P7’s and P6’s Answers Regarding the Influence of Eye-Tracking

[Translation: “(1) *Affected. I could hear some machine operation sounds, especially when I moved my gaze over a large distance or when I finished reading one line and started the next. This variation in sound was more noticeable during the first article. It seemed to prompt me to go back and reread what I had read a second ago.* (2) *No major impact. Except that being told about the tracking unconsciously created psychological pressure, leading me to lower my reading speed and reduce my range of eye movements. The heightened concentration made me feel somewhat fatigued by the end of each article.*” ]

[Translation: “(1) *Gap-filling processes: Affected, aware of being eye-tracked, I tried not to stay too long on a sentence or a word, not to look away from the screen, not to look elsewhere and think for a long time.* (2) *The accuracy of gap-filling: Not affected.*”]
